# Facial Prurigo Pigmentosa After Bariatric Surgery

**DOI:** 10.7759/cureus.6909

**Published:** 2020-02-07

**Authors:** Musaed M Alsebayel, Yasser A Ghobara, Ahmed Al-Issa

**Affiliations:** 1 Dermatology, King Saud Bin Abdul-Aziz University for Health Sciences, Riyadh, SAU; 2 Dermatology, Derma Clinic, Riyadh, SAU

**Keywords:** prurigo pigmentosa, face, bariatric surgery, ketosis

## Abstract

Prurigo pigmentosa (PP) is a rare, inflammatory, idiopathic skin disorder, which typically presents as symmetrically scattered pruritic erythematous reticulated papules with occasional vesicles. PP has been primarily a disease of the trunk and the neck, and, to the authors’ knowledge, there has been only two reported cases of PP where the forehead/facial areas were involved worldwide. Interestingly, there have not been any reported cases of after bariatric surgery PP with facial involvement. Herein, we present a case of PP after laparoscopic sleeve gastrectomy with involvement of the face, chest, and back.

## Introduction

Prurigo pigmentosa (PP) is a rare, inflammatory, idiopathic skin disorder, which typically presents as symmetrically scattered pruritic erythematous reticulated papules with occasional vesicles primarily affecting the neck, back, and chest. It is classically seen in young women of Japanese origin and was first described by Nagashima et al. in 1971 [1-3]. The pathogenesis of PP is not fully understood; however, several factors have been associated with the condition such as diabetes mellitus, fasting/diet, pregnancy, atopy, and being in a ketotic state [4-8]. A few recent studies have shown a growing association between PP and bariatric surgery, which may be due to the metabolic disturbances seen as a result of such procedure [9-11]. Increased physician awareness about post-bariatric PP is much needed, especially with the growing popularity of bariatric and weight reduction procedures. PP has been primarily a disease of the trunk and the neck, and, to the authors’ knowledge, there has been only two reported cases of PP where the forehead/facial areas were involved worldwide [12,13]. Interestingly, there have not been any reported cases of after bariatric surgery PP with facial involvement. Herein, we present a case of PP after laparoscopic sleeve gastrectomy with involvement of the face, chest, and back.

## Case presentation

**Figure 1:**
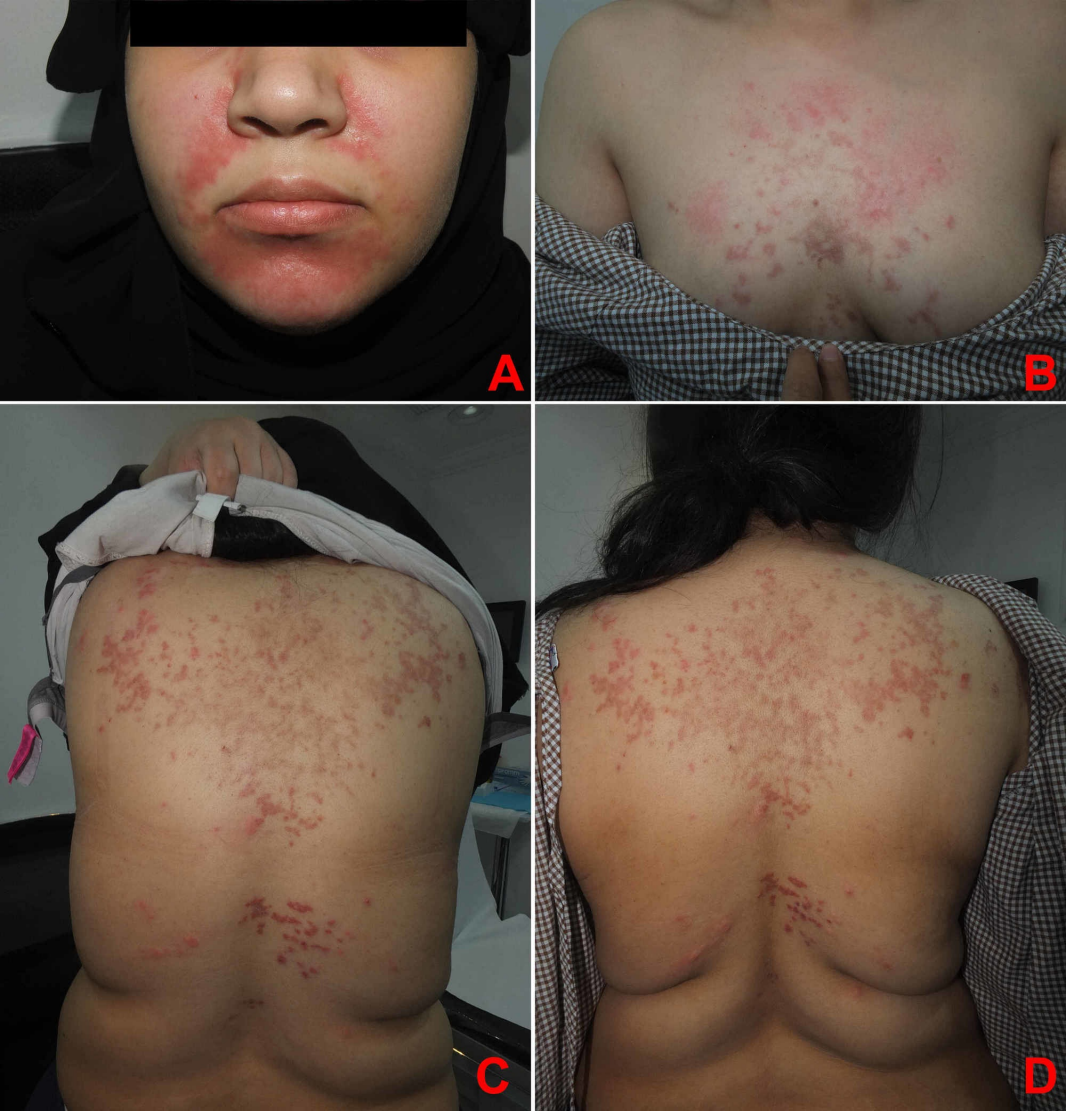
(A) Face. (B) Chest. (C, D) Back. Multiple erythematous reticulated papules and plaques with hyperpigmented macules and patches.

**Figure 2:**
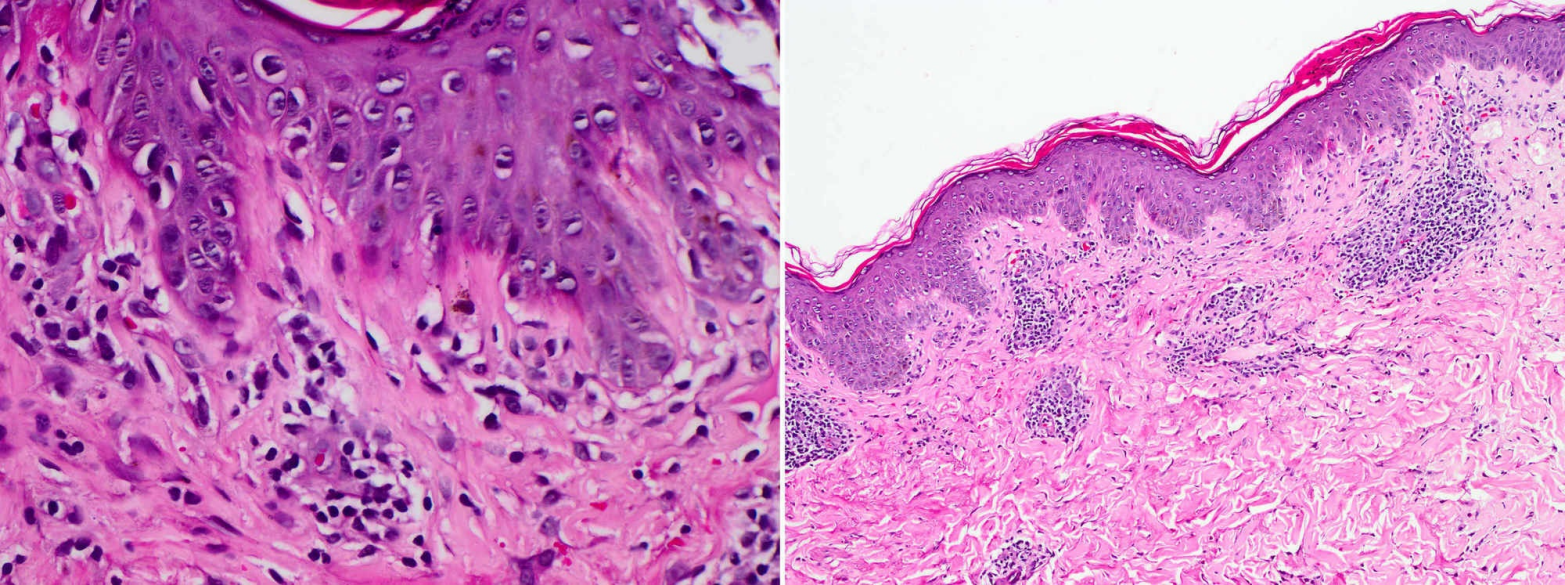
Mild vacuolar basal cell changes with focal lymphocytic exocytosis and scale crusting, in addition to moderate superficial perivascular lymphocyte infiltrate with a few neutrophils, eosinophils, and extraverted red blood cells.

**Figure 3:**
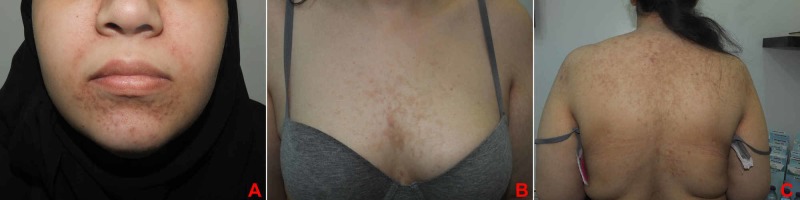
(A) Face. (B) Chest. (C) Back. Significant improvement leaving post-inflammatory hyperpigmentation.

An otherwise healthy 25-year-old single female presented with a 10-day history of itchy erythematous papules over the face, chest, and upper back, which started three days after laparoscopic sleeve gastrectomy with no intra/post-operative complications. The patient was on daily multivitamin supplements (fusion chewable tablets), daily biotin, and vitamin D (50,000 IU, weekly for eight weeks). She denied taking any other medication or being currently on a ketogenic diet. The patient has lost 7 kg since the operation, and weight at presentation was 106 kg. There was a past history of a similar eruption four years ago, two weeks after following a ketogenic diet, which spontaneously resolved after resumption of her normal diet. Physical examination revealed multiple erythematous reticulated papules and plaques with hyperpigmented macules involving the face, the chest, and the upper back/shoulder (Figure 1). A skin punch biopsy was obtained from the left shoulder which showed mild vacuolar basal cell changes with focal lymphocytic exocytosis and scale crusting. There was moderate superficial perivascular lymphocyte infiltrate with few neutrophils, eosinophils, and extraverted red blood cells. Histological features were consistent with the diagnosis of PP (Figure 2). Considering the clinical findings in addition to the histopathology results, a diagnosis of PP was established. The patient was prescribed minocycline extended-release tablets (80 mg, QD for one month) and topical mometasone furoate cream (BID for one week). A four-week follow-up showed complete resolution of the eruption, with post-inflammatory hyperpigmentation (Figure 3).

 

## Discussion

The term “prurigo pigmentosa” was first mentioned in the literature in 1978, seven years after its first description by Nagashima et al. [1,14]. PP is most commonly seen in the third decade of life, with a female-to-male ratio as high as 6:1, and has been previously described in more than 300 cases, largely affecting the Japanese and East-Asian populations [15-18]. However, increasing reports of PP have been observed in the Western countries, and, more recently, in the Middle East, which might be reflecting the increased awareness about the condition [9-11,15].

The etiology and pathogenesis of prurigo pigmentosa are not fully clear; however, several factors have been strongly associated with the condition, which may be grouped as metabolic (diabetes, ketosis), hormonal (pregnancy), and physical factors (skin friction, trauma, acupuncture) [2,3]. Elevated ketone bodies have been documented in several patients with PP, which is hypothesized to cause distribution of ketone bodies around blood vessels, leading to perivascular inflammation, mainly neutrophilic. This may explain the excellent response seen in patients treated with anti-neutrophilic agents such as dapsone and tetracyclines [2,3,10,11].

Ketosis-associated PP has been observed in several reports, which have been typically a result of insulin-dependent diabetes, anorexia nervosa, and dieting [2,3,6,7]. However, only three recent articles have reported an association between bariatric surgeries and PP [9-11]. This may be explained by the decreased serum insulin levels and food intake, and possible elevation of ketone bodies after the procedure, resulting in a ketotic state [19,20]. It is conceivable that the low number of reports of PP after bariatric surgery could be owing to under-recognition and underdiagnosis. Especially with the growing popularity of such procedures, more light should be shed on this rare complication to increase physicians’ awareness about the disease.

PP has always been primarily a disease of the neck and the trunk. Hair, nails, and mucous membrane involvement have never been reported. Facial/forehead involvement of the disease has been previously described in only two cases in 1983 and 1984, none of which were associated with bariatric surgery [12,14]. Facial PP, though extremely unusual, can be of special importance, due to its potential psychological impact on patients. Thus, it is important to consider facial PP in the presence of the clinical and historical findings in order to achieve early diagnosis, treatment, and future complications of this condition.

Tetracycline antibiotics have been the mainstay of treatment of PP, which may be due to their anti-inflammatory effect on neutrophilic chemotaxis, migration, and function [10,11]. Other therapeutic agents such as macrolide antibiotics, dapsone, potassium iodide, and low-dose isotretinoin have also been used, with varying efficacy [9,18]. Topical and systemic corticosteroids and antihistamines have a limited role in the treatment of PP [15,17]. Resumption of normal diet and improvement of oral intake in cases of ketosis were also found to be helpful in the treatment of the disease [3]. Table 1 summarizes and compares previous cases in the literature with our case.

**Table 1:** Comparing previously reported post-bariatric surgery cases to our case [9,10].

Prurigo Pigmentosa After Bariatric Surgery			
	Abbass et al.	Al-Dawsari et al.	Our case
Sex	Female	Female	Female
Age	40 years	37 years	25 years
Ethnicity	Middle Eastern	Middle Eastern	Middle Eastern
Onset post-surgery	7 days	12 days	3 days
Site	Back, chest	Back, chest	Face, upper back, chest
Skin examination	Erythematous papules and vesicles, with reticulated hyperpigmented macules	Pruritic erythematous reticulated papules and plaques, with a few urticarial plaques and hyperpigmented patches	Multiple erythematous reticulated papules and plaques with hyperpigmented macules
Histopathology	Focal epidermal parakeratosis with minimal spongiosis; mild epidermal hyperplasia; mild/moderate dense superficial and mid-dermal perivascular and interstitial lymphoneutrophilic infiltrate with scattered eosinophils	Focal epidermal parakeratosis with minimal spongiosis; mild epidermal hyperplasia; superficial and mid-dermal perivascular lymphocytic cuffing with rare eosinophils	Mild vascular basal cells changes with focal lymphocytic exocytosis and scale crusting; moderate superficial perivascular lymphocyte infiltrate with few neutrophils, eosinophils, and extraverted red blood cells
Treatment	Doxycycline (100 mg BID)	IV and oral corticosteroids; minocycline	Minocycline (80 mg, QD); topical mometasone furoate cream (BID)
Follow-up	Significantly improved	No response to steroid therapy; minocycline not tolerated; spontaneous improvement	Significant improvement leaving post-inflammatory hyperpigmentation

## Conclusions

With the growing popularity of bariatric surgery leading to ketotic state, it is possible that an increasing number of PP may be seen. Especially with facial PP, physicians should have a high index of suspicion, as many cases are under-recognized and underdiagnosed. Awareness of the condition is crucial in order to achieve early recognition, diagnosis, and treatment, and to prevent disease progression and future complication.

## References

[1] Nagashima M, Ohshiro A, Shimizu N (1971). A peculiar dermatosis with gross reticular pigmentation [in Japanese]. Jpn J Dermatol.

[2] Oh YJ, Lee MH (2012). Prurigo pigmentosa: a clinicopathologic study of 16 cases. J Eur Acad Dermatol Venereol.

[3] Böer A, Misago N, Wolter M, Kiryu H, Wang XD, Ackerman AB (2003). Prurigo pigmentosa: a distinctive inflammatory disease of the skin. Am J Dermatopathol.

[4] Kwon HJ, Kim MY, Kim HO, Park YM (2006). Two cases of prurigo pigmentosa in atopic patients. J Dermatol.

[5] Kubota Y, Koga T, Nakayama J (1998). Bullous prurigo pigmentosa and diabetes. Eur J Dermatol.

[6] Teraki Y, Teraki E, Kawashima M, Nagashima M, Shiohara T (1996). Ketosis is involved in the origin of prurigo pigmentosa. J Am Acad Dermatol.

[7] Kobayashi T, Kawada A, Hiruma M, Ishibashi A, Aoki A (1996). Prurigo pigmentosa, ketonemia and diabetes mellitus. Dermatology.

[8] Leone L, Colato C, Girolomoni G Prurigo pigmentosa in a pregnant woman. Int J Gynaecol Obstet.

[9] Al-Dawsari NA, Al-Essa A, Shahab R, Raslan W (2019). Prurigo pigmentosa following laparoscopic gastric sleeve. Dermatol Online J.

[10] Abbass M, Abiad F, Abbas O (2015). Prurigo pigmentosa after bariatric surgery. JAMA Dermatol.

[11] Alshaya MA, Turkmani MG, Alissa AM (2019). Prurigo pigmentosa following ketogenic diet and bariatric surgery: a growing association. JAAD Case Rep.

[12] Miyakawa S, Kurihara S, Nishikawa T (1984). Prurigo pigmentosa affecting the forehead. Dermatologica.

[13] Oohara K (1983). Prurigo pigmentosa initiating on the face. Rinsho Dermatol.

[14] Nagashima M (1978). Prurigo pigmentosa: clinical observations of our 14 cases. J Dermatol.

[15] Hijazi M, Kehdy J, Kibbi AG, Ghosn S (2014). Prurigo pigmentosa: a clinicopathologic study of 4 cases from the Middle East. Am J Dermatopathol.

[16] Gironi LC, Farinelli P, Giacalone A, Colombo E (2015). The efficacy of minocycline in inflammatory dermatoses: a case of prurigo pigmentosa of prepubescent onset in Western world. Dermatol Ther.

[17] Michaels JD, Hoss E, DiCaudo DJ, Price H (2015). Prurigo pigmentosa after a strict ketogenic diet. Pediatr Dermatol.

[18] Beutler BD, Cohen PR, Lee RA (2015). Prurigo pigmentosa: literature review. Am J Clin Dermatol.

[19] Halawi A, Abiad F, Abbas O (2013). Bariatric surgery and its effects on the skin and skin diseases. Obes Surg.

[20] Valkenborgh T, Bral P (2013). Starvation-induced ketoacidosis in bariatric surgery: a case report. Acta Anaesthesiol Belg.

